# Persistent Cardiac Magnetic Resonance Imaging Features of Myocarditis Detected Months After COVID-19 Infection

**DOI:** 10.7759/cureus.14250

**Published:** 2021-04-01

**Authors:** Basel Abdelazeem, Mariem Borcheni, Saed Alnaimat, Sagar Mallikethi-Reddy, Abdulbaset Sulaiman

**Affiliations:** 1 Department of Internal Medicine, McLaren Health Care, Flint/Michigan State University, Flint, USA; 2 Department of Internal Medicine, Sfax Faculty of Medicine, Sfax, TUN; 3 Department of Cardiology, McLaren Health Care, Flint/Michigan State University, Flint, USA

**Keywords:** covid-19, sars-cov2, myocarditis, cardiac magnetic resonance, echocardiography

## Abstract

Acute myocarditis is commonly caused by viral infections resulting from viruses such as adenovirus, enteroviruses, and, rarely, coronavirus. It presents with nonspecific symptoms like chest pain, dyspnea, palpitation, or arrhythmias and can progress to dilated cardiomyopathy or heart failure. Fulminant myocarditis is a potentially life-threatening form of the condition and presents as acute, severe heart failure with cardiogenic shock.

In this report, we discuss a case of a 41-year-old female who presented with cough and chest pain of two days' duration. The patient had a new-onset atrial flutter. Her chest auscultation revealed bilateral crackles. Laboratory workup revealed elevated troponin levels, and the patient tested positive for coronavirus disease 2019 (COVID-19) by nasopharyngeal swab polymerase chain reaction (PCR). Transthoracic echocardiogram revealed a low left ventricular (LV) ejection fraction of 35-40% compared to 55% one year prior, as well as a granular appearance of LV myocardium. The patient's condition subsequently improved clinically and she was discharged home. Due to cardiac involvement and characteristic myocardial appearance on the echocardiogram, cardiac magnetic resonance (CMR) imaging was performed for further evaluation about two months from the date of admission. CMR showed extensive myocardial inflammation with a typical pattern of sub-epicardial and mid-wall delayed enhancement, confirming the diagnosis of myocarditis.

This case highlights myocarditis as a potential complication of COVID-19 that requires early diagnosis and proper management to improve patients' quality of life. Additionally, we highlight the features of myocarditis on CMR in the acute phase and two months after clinical recovery.

## Introduction

Acute myocarditis varies in its presentation, ranging from mild symptoms to life-threatening conditions. Fulminant myocarditis is an uncommon complication. It is characterized by sudden and severe diffuse cardiac inflammation, leading to cardiogenic shock, ventricular arrhythmias, or death from multiorgan system failure [1,2].

Acute myocarditis is a rare disease, with a reported annual global incidence of 22 cases per 100,000 population and a mortality rate of 25-56% within 3-10 years [3,4]. Myocarditis can occur in 1-5% of patients with acute viral infections. Myocardial injury can be associated with severe acute respiratory syndrome coronavirus 2 (SARS-CoV-2) infection and occurs in up to 7-23% of the cases [2]. Usually, it affects patients over 50 years of age with a slight predominance among males (58% vs. 42%) [5]. The majority of patients do not have any previously known comorbidities (50%), but among those with a past medical history, hypertension has been the most commonly reported comorbidity [6].

The diagnosis of acute myocarditis is based on clinical presentation, serum biomarkers, and echocardiography. Cardiac magnetic resonance (CMR) imaging is a valuable diagnostic tool for this condition. It can show features of acute myocarditis not only in the acute phase but also after recovery. In this report, we present a case of a 41-year-old female who was found to have acute myocarditis as a complication of coronavirus disease 2019 (COVID-19) with persistent CMR features detected months after recovery. This case highlights the importance of CMR imaging in COVID-19 patients with symptoms suggestive of cardiac involvement.

## Case presentation

A 41-year-old female with a past medical history of hypertension, type 2 diabetes mellitus, and previous non-ST-elevation myocardial infarction with normal coronary arteries (MINOCA) three years prior presented with symptoms of sharp chest pain, dry cough, and shortness of breath, which had started two days prior to the presentation. Her vital signs were remarkable for a blood pressure of 122/91 mmHg, heart rate of 180 beats per minute with an irregular rate, a temperature of 36.4 °C, respiratory rate of 24 breaths per minute, and an oxygen saturation of 98% on 5 L nasal cannula. On examination, the patient appeared to be in mild distress. No jugular venous distention was appreciated. Her chest auscultation revealed bilateral crackles.

**Figure 1:**
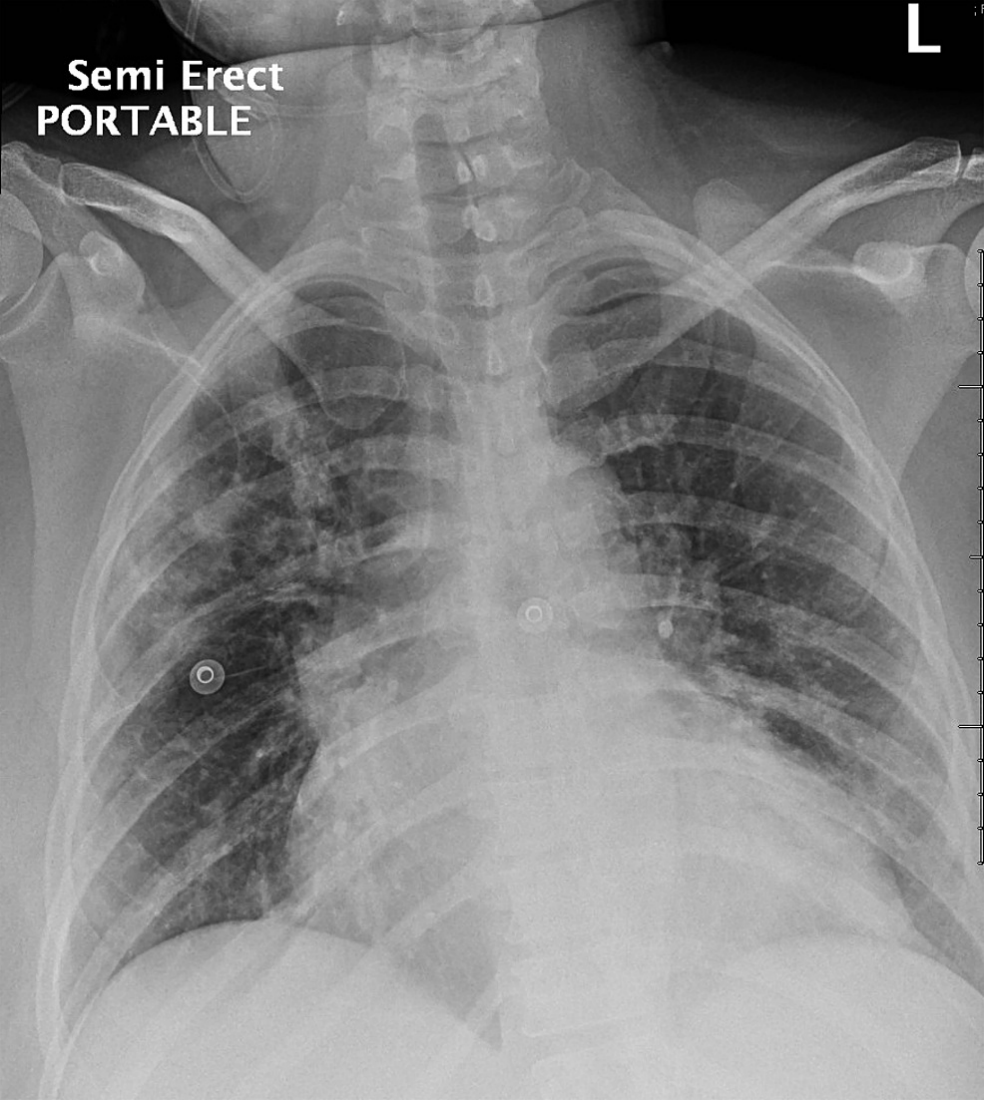
Chest X-ray of the patient Chest X-ray revealed patchy bilateral peripheral lung infiltrate. No pleural effusion pneumothorax is seen

**Figure 2:**
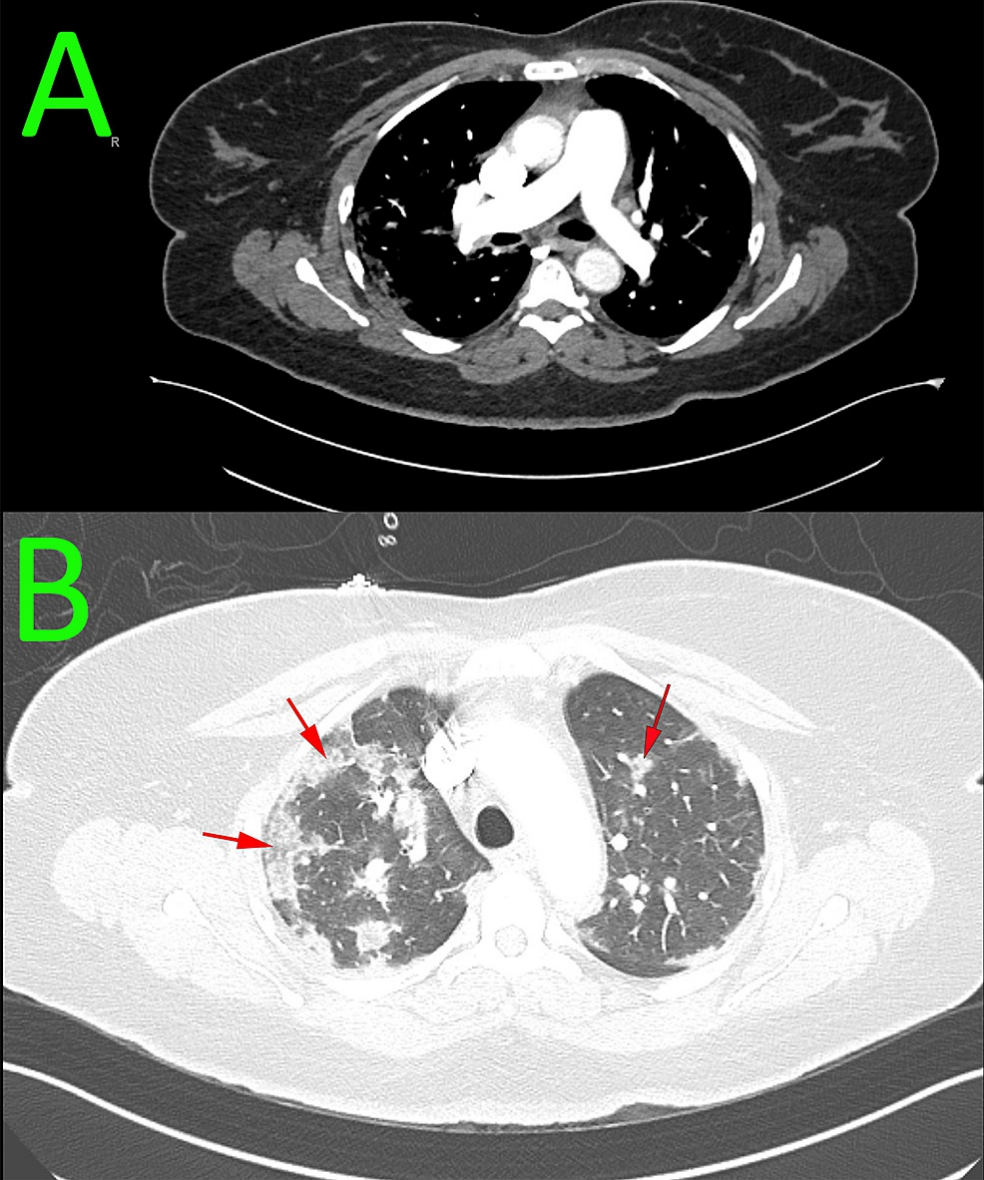
CT angiogram of the chest A: the image revealed no filling defect seen in the central or segmental pulmonary arterial branches to suggest pulmonary thromboembolism. B: the image demonstrated moderate bilateral patchy peripheral ground-glass opacities (red arrows) CT: computed tomography

**Figure 3:**
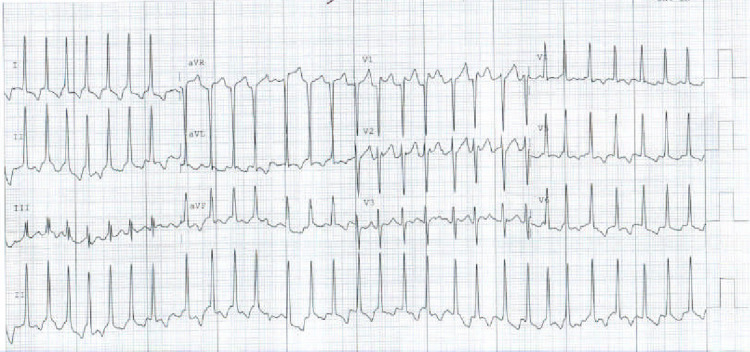
EKG on the day of admission showing atrial flutter with a rapid ventricular response EKG: electrocardiogram

**Figure 4:**
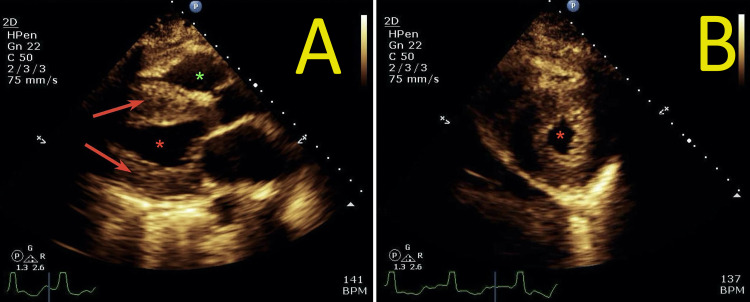
Transthoracic echocardiogram performed on the second day of admission Parasternal long-axis view (panel A) and parasternal short-axis view (panel B) Transthoracic echocardiogram demonstrated concentric left ventricular hypertrophy with the characteristic granular appearance of the myocardium. The red arrow indicates left ventricular myocardium. The red asterisk indicates the left ventricular cavity. The green asterisk indicates the right ventricular cavity

The patient's laboratory workup is summarized in Table 1. A nasopharyngeal swab polymerase chain reaction (PCR) was positive for COVID-19 and blood cultures showed no growth. Chest X-ray revealed patchy bilateral peripheral lung infiltrates (Figure 1), and CT angiography of the chest showed no pulmonary embolism but revealed bilateral patchy ground-glass lung infiltrates consistent with COVID-19 pneumonia (Figure 2). EKG showed a new atrial flutter with a rapid ventricular response (Figure 3). Transthoracic echocardiogram revealed a new low left ventricular (LV) ejection fraction of 35-40%, with LV hypertrophy, as well as a granular appearance of LV myocardium suspicious for infiltrative cardiomyopathy (Figure 4). Echocardiogram obtained one year prior had shown LV ejection fraction of 50-55% with LV hypertrophy.

**Table 1:** Laboratory workup of the patient WBC: white blood cells; BNP: B-type natriuretic peptide; TSH: thyroid-stimulating hormone

Labs	Values at admission	Reference range
WBC	8.5 x 10^3^/uL	4.5-11 x 10^3^/uL
Hemoglobin	14.8 g/dl	12-15.7 g/dl
Platelets	243 x 10^3^/uL	140-440 x 10^3^/uL
Troponin I	0.67 ng/mL	0.0000-0.0400 ng/mL
BNP	218 pg/mL	2-100 pg/mL
D-dimer	0.97 mg/L	0.19-0.52 mg/L FEU
Interleukin-6	2.7 pg/mL	0-5 pg/ml
TSH	0.480 uIU/mL	0.350-5.500 uIU/mL

The patient was started on intravenous (IV) diltiazem drip, but her heart rate was difficult to control; hence she was started on IV amiodarone and eventually converted to sinus rhythm. She was started on IV heparin for anticoagulation. Her respiratory status deteriorated, and she was subsequently intubated and mechanically ventilated. The COVID-19 pneumonia was treated with a combination of steroids, antibiotics, remdesivir, and convalescence plasma. She improved clinically and was discharged home on oral amiodarone, metoprolol succinate, and warfarin.

**Figure 5:**
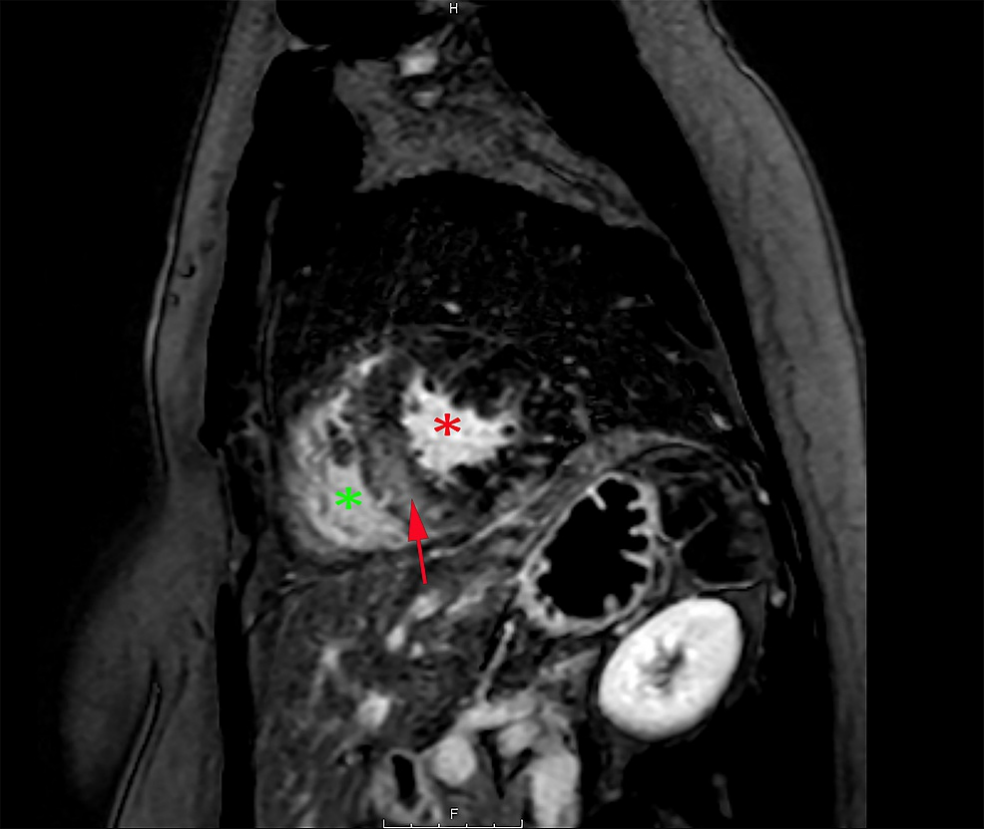
CMR imaging short-axis view CMR image showing extensive myocarditis with a typical pattern of delayed enhancement involving sub-epicardial and mid-wall of the left ventricular and interventricular septum (red arrow). The red asterisk indicates the left ventricular cavity. The green asterisk indicates the right ventricular cavity CMR: cardiac magnetic resonance

CMR obtained about two months from the date of admission showed extensive myocarditis with a typical pattern of sub-epicardial and mid-wall delayed enhancement (Figure 5). There was no edema to indicate the acuteness of the disease. LV systolic function was normal with an ejection fraction of 60-65%. These findings confirmed that the patient had suffered from an acute illness at the time of her initial presentation, causing acute symptoms, a drop in LV ejection fraction, and the development of new arrhythmia, which had all resolved by the time of CMR acquisition.

## Discussion

Acute myocarditis is diagnosed based on clinical presentation, serum biomarkers, and echocardiography. Patients with acute myocarditis have elevated inflammatory markers such as C-reactive protein, erythrocyte sedimentation rate, and procalcitonin. Cardiac enzymes including troponin, B-type natriuretic peptide (BNP), and N-terminal pro-B-type natriuretic peptide (NT-proBNP) can be elevated in viral myocarditis. Negative serial troponin levels make the diagnosis of myocarditis less likely [2]. On the other hand, high troponin levels can be associated with worse prognosis and higher in-hospital mortality. EKG changes are nonspecific and found to be highly variable, including nonspecific ST-segment changes (which occur in up to 71% of the cases), T-wave abnormalities, sinus tachycardia, and conduction abnormalities (bundle branch blocks/atrioventricular conduction delays).

Echocardiography is nonspecific for diagnosing myocarditis, although it may demonstrate LV regional or global dysfunction and occasionally pericardial effusion. Echocardiography helps exclude valvular or other cardiac causes of clinical presentation. However, echocardiography may play a role in classifying myocarditis. It can show increased wall thickness in fulminant myocarditis, ventricular chamber dilation, and normal wall thickness in less severe myocarditis [2,7]. Additionally, the granular or sparkling appearance of the myocardium on echocardiography has been reported in cases of myocarditis with severe fibrosis, hypertrophic cardiomyopathy, and other infiltrative diseases of the myocardium such as cardiac amyloidosis [7]. In our case, echocardiography showed a new low LV ejection fraction of 35-40%, with concentric LV hypertrophy, as well as a granular appearance of LV myocardium suspicious for infiltrative cardiomyopathy. No pericardial effusion or significant valvular abnormalities were seen.

The gold standard method for the diagnosis of myocarditis is an endomyocardial biopsy. However, risks associated with the procedure, including contagious spread risk and its false-negative rate, make the diagnostic value of this procedure much less favorable. Since its advent, CMR has played a central role in cardiovascular diagnostics because of its spatial resolution, quantitative accuracy, and interobserver consistency.

CMR can provide unique insights into tissue-level pathologies of myocarditis, such as myocardial edema and fibrosis. Late gadolinium enhancement (LGE) of subepicardial myocardium is highly suggestive of myocarditis, mainly when the abnormality is limited to this zone and not following a vascular territory. LGE indicates necrosis in the acute setting and scar at a chronic stage. Identifying acute myocardial inflammation on CMR is based on the presence of edema, capillary leak, and necrosis/scar on two out of three imaging sequence techniques, including T2-weighted imaging, early gadolinium enhancement, and LGE (the Lake Louise criteria) [8,9]. Two recent meta-analyses have revealed that using the Lake Louise criteria or its individual component has a similar diagnostic accuracy [10,11], but full Lake Louise criteria have a higher positive predictive value [12].

CMR has a diagnostic accuracy of up to 80%, making it capable of ruling in myocarditis and differentiating ischemia from non-ischemic cardiomyopathies [8]. However, CMR is limited by availability, cost, prolonged acquisition time, some breath-holding requirements, and other technical requirements. Current expert and medical societies' consensus recommends performing CMR in COVID-19 patients who present with symptoms suggestive of cardiac involvement [13].

Our case highlights the importance of CMR as a diagnostic and prognostic tool in acute myocarditis. CMR obtained about two months from the date of admission showed extensive myocarditis with a typical pattern of sub-epicardial and mid-wall delayed enhancement, but no myocardial edema was present. Usually, CMR features of acute myocarditis are present in the acute phase and persist after recovery. Our patient was clinically diagnosed with myocarditis and confirmed by a drop in LV ejection fraction and new arrhythmia development. The patient was found to have persistent LGE with a typical distribution pattern two months after the initial diagnosis of myocarditis despite an apparent clinical improvement, which is a unique finding.

Other diagnostic modalities include contrast-enhanced cardiac CT and nuclear imaging techniques such as technetium-99m-MIBI or thallium-201 single-photon emission CT imaging [14]. The usage of contrast and radiation is a limiting factor. Also, those modalities are less specific to myocarditis [15,16].

## Conclusions

Acute myocarditis is a rare and fatal complication of COVID-19. We reported a case of a 41-year-old female who initially presented with cough and chest pain and was subsequently diagnosed with myocarditis secondary to COVID-19, which was confirmed by CMR. An awareness about CMR features is crucial for early diagnosis and proper management to improve patients' quality of life. Myocardial involvement is not only seen in the acute phase of COVID-19 but can also be detected months after recovery. This further highlights the significance of CMR imaging in COVID-19 patients with symptoms suggestive of cardiac involvement.
